# Is a Combination of Melatonin and Amino Acids Useful to Sarcopenic Elderly Patients? A Randomized Trial

**DOI:** 10.3390/geriatrics4010004

**Published:** 2018-12-24

**Authors:** Mariangela Rondanelli, Gabriella Peroni, Clara Gasparri, Vittoria Infantino, Mara Nichetti, Giovanni Cuzzoni, Daniele Spadaccini, Simone Perna

**Affiliations:** 1 IRCCS Mondino Foundation, 27100 Pavia, Italy; mariangela.rondanelli@unipv.it; 2 Department of Public Health, Experimental and Forensic Medicine, Unit of Human and Clinical Nutrition, University of Pavia, 27100 Pavia, Italy; gabriella.peroni01@universitadipavia.it (G.P.); clara.gasparri01@universitadipavia.it (C.G.); viriainfantino@hotmail.it (V.I.); dietista.mara.nichetti@gmail.com (M.N.); daniele.spadaccini01@universitadipavia.it (D.S.); 3 Department of Biomedical Science and Human Oncology, University of Bari, 70121 Bari, Italy; 4 A.S.P.S. Margherita Geriatric Department, 27100 Pavia, Italy; giovanni_cuzzoni@asppavia.it; 5 Department of Biology, College of Science, University of Bahrain, Sakhir Campus P.O. Box 32038, Kingdom of Bahrain

**Keywords:** melatonin, sarcopenia, muscle mass, inflammation, antioxidants

## Abstract

This study evaluated the effectiveness of a 4-week intervention of melatonin and essential aminoacid supplementation on body composition, protein metabolism, strength and inflammation in 159 elderly sarcopenic patients (42/117, men/women), assigned to four groups: isocaloric placebo (P, n = 44), melatonin (M, 1 mg/daily, n = 42), essential amino acids (eAA 4 g/daily, n = 40) or eAA plus melatonin (eAAM, 4 g eAA and 1 mg melatonin/daily, n = 30). Data from body composition (dual X-ray absortiometry (DXA)), strength (handgrip test) and biochemical parameters for the assessment of protein metabolism (albumin) and inflammation (CRP) were collected at baseline and after the 4-week intervention. Compared with P and M, supplementation with eAA plus M increased total fat-free mass (vs. P: +2190 g; *p* < 0.01; vs. M: +2107 g; *p* < 0.05). M alone lowered albumin levels (vs. P: −0.39 g; *p* < 0.01; vs. eAA: −0.47 g; *p* < 0.01). This data on albumin was confirmed by within-group analysis (M −0.44g; *p* < 0.001; eAAM: −0.34 *p* < 0.05). M and eAA seemed to lower the percentage of gynoid fat (*p* < 0.05) and android fat (*p* < 0.01). No significant changes in inflammation or strength were reported. A 4-week intervention with eAA plus M together may be effective in enhancing fat-free-mass compared to M and P but not versus eAA. M alone demonstrates a negative effect on albumin level.

## 1. Introduction

Sarcopenia is a progressive, insidious process characterized by the reduction of lean muscle mass [1], and is the most common disorder affecting the aging population over sixty [2]. Sarcopenia can be managed with multidimensional approaches that include physical training, nutritional support and metabolic and anabolic treatment [3]. Oral supplementation with essential amino acids (eAAs) stimulates muscle protein synthesis in the elderly [4]. Decreased melatonin levels are associated with the aging process and age-related conditions. In fact, melatonin levels in urine are inversely associated with sarcopenia in postmenopausal women [5].

In addition, evidence suggests that melatonin may be as effective as testosterone in the prevention of muscle atrophy in animal models through the IGF-1 axis [6]. Moreover, melatonin significantly attenuated TNF-α-induced ROS generation and apoptosis [7]. In addition, decreased muscle fiber diameter and increased muscle cell proteolysis by TNF-α was highly attenuated by treatment with melatonin [8]. As well as improving muscle function, melatonin decreased plasma creatine kinase activity, a marker for muscle injury, increased total glutathione content and lowered the oxidized/reduced glutathione ratio, indicating better redox status of the muscle [9]. Melatonin is also able to restore impaired contractility of the detrusor muscle through the normalization of Ca^2+^-dependent and -independent contraction, mitochondrial polarity, neuromuscular function and oxidative stress [10]. Recent findings strongly suggest that melatonin administration also significantly reduces the hyperoxidative and inflammatory process in Duchenne muscular dystrophy (DMD) patients, reducing muscle degeneration [11].

Likewise, melatonin lowers chronic inflammation levels and reduces vascular aging, all of which are usually present in sarcopenic muscle tissue. Similarly, melatonin improves endocrine signaling, which deteriorates in aged individuals. As a consequence, melatonin may be useful in preventing or treating sarcopenia-associated diseases, including osteoporosis and neuromuscular dysfunction [12].

Against this background, it is intriguing that Tuomi et al. showed that a common (about 30%) human type 2 diabetes (T2D) risk variant of the melatonin receptor 1B gene affects insulin release [13]; this study demonstrated that melatonin treatment inhibits insulin secretion, with at-risk carriers exhibiting higher glucose levels. Moreover, various authors have found that a variant of the MTNR1B gene is associated with elevated plasma glucose levels, a reduction of the early insulin response to both oral and intravenous glucose, faster deterioration of insulin secretion over time, and increased future risk of T2D [14,15,16]. This association has subsequently been confirmed in other populations [17,18,19].

So, if melatonin exerts an inhibitory effect on insulin secretion [20,21], and many studies suggest that amino acids and insulin play major roles in promoting postprandial protein anabolism [22,23], the hypothesis arises that melatonin intake could have a detrimental effect on protein metabolism through the inhibition of insulin secretion, particularly in the case of sarcopenia, which has repeatedly been shown to be associated with resistance of muscle tissue to the anabolic effects of insulin [24].

As the action of melatonin on protein metabolism and muscle mass has been debated in the literature and no clinical intervention trials which study the activity of melatonin in patients with sarcopenia have been published, the aim of this preliminary study is to compare the effect on protein metabolism (albumin), body composition (fat free mass, fat mass, android and gynoid mass, assessed by dual X-ray absortiometry (DXA)), muscle strength and inflammation (C-reactive protein) in sarcopenic elderly subjects in 4 different study groups: 1. placebo, 2. melatonin, 3. essential amino acids, 4. melatonin in combination with amino acids.

## 2. Materials and Methods

### 2.1. Setting

The study was conducted in the city of Pavia (Italy). We evaluated elderly women and men consecutively admitted to our physical medicine and rehabilitation division (Santa Margherita Institute). Patients in our hospital are either referred from acute care hospitals in the region of Lombardy (in northern Italy) for follow-up care and/or rehabilitation, or are local residents with chronic conditions associated with age, requiring revision and updating of their treatments.

### 2.2. Study Population

Eligible patients were aged 65 years or older and sarcopenic, defined as suffering loss of muscle mass as assessed by DXA (Skeletal Muscle Index [SMI] was <7.23 kg/m^2^ in men and <5.45 kg/m^2^ in women) [25] and loss of strength, evaluated by dynamometer and defined as <30 kg for men and <20 kg for women, using the average value of the two handgrip measurements of the dominant hand [26]. 

**Figure 1:**
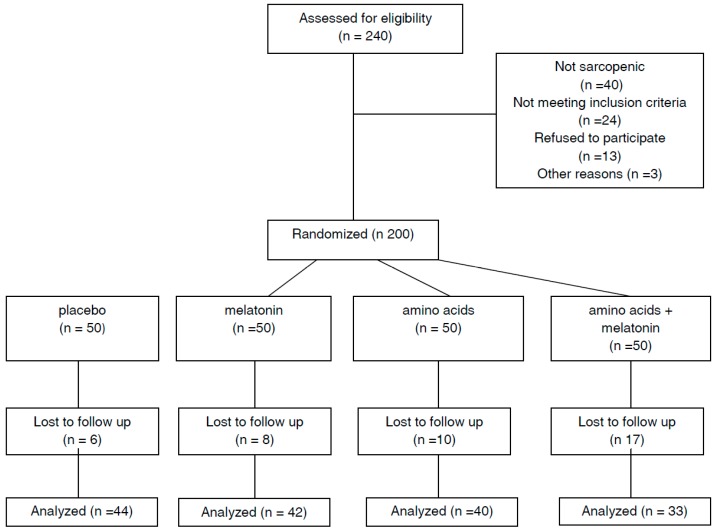
Flow diagram of the study.

Subjects who were not affected by acute illness, severe liver, heart or kidney dysfunction, or severe dementia and had a bodyweight that had been stable for 6 months were included in the study. Moreover, subjects with uncontrolled diabetes, dysthyroidism and other endocrinopathies, or neoplasia, or patients treated with steroids or entirely unable to walk were excluded. The study was conducted in accordance with the Declaration of Helsinki and the study design was approved by the ethics committee of the University of Pavia and individual written informed consent was obtained from each participant. Data were gathered from the end of January 2014 to the end of June 2016. The participants’ flow diagram is shown in Figure 1.

### 2.3. Observed Outcome Variables

Body composition assessment: body composition (FFM, fat mass, gynoid and android fat distribution) was measured by DXA with the use of a Lunar Prodigy DXA (GE Medical Systems). The in vivo coefficient of variations were 0.89% and 0.48% for whole-body fat (fat mass) and FFM, respectively. The Skeletal Muscle Index (SMI) was taken as the sum of the fat-free soft tissue mass of arms and legs divided by height^2^, following the Rosetta Study Cutoff (SMI: <7.23 kg/m^2^ in men and 5.45 kg/m^2^ in women) [25].

The evaluations of body composition by DXA were performed before supplementation and after 1 month.

Body weight was measured to the nearest 0.1 kg using a precision scale, with the subjects wearing light clothing and no shoes, using a standardized technique, and BMI was calculated (kg/m^2^).

#### 2.3.1. Assessment of Functional Performance

Handgrip strength was assessed using a Jamar dynamometer adhering to the standardized protocol recommended by the American Society of Hand Therapists. Dominant and non-dominant handgrip strength was measured with a calibrated dynamometer (Baseline, Elmsford, NY, USA). A weak handgrip was defined as <30 kg for men and <20 kg for women, based on the average value of the two handgrip measurements of the dominant hand [26].

#### 2.3.2. Blood Sample Measurements

Fasting venous blood samples were drawn between 8 a.m. and 10 a.m., with the subjects in a sitting position. Blood handling and collection were carried out under strictly standardized conditions and in line with manufacturers’ recommendations. High performance liquid chromatography was used to measure total plasma homocysteine levels. Serum albumin was also analyzed using a nephelometric method, with a 2% coefficient of variation: this marker lacks the sensitivity to assess protein malnutrition in the short term because of its long turnover rate (18 days), so although its levels remained one of the reference standards for the diagnosis of protein malnutrition, they are a somewhat tarnished standard [27]. It has been used for its easy availability and because it is routinely measured, especially in elderly hospitalized patients. Fasting blood total cholesterol and triglyceride levels were measured by an automatic biochemical analyzer. High-sensitivity C-reactive protein (CRP), erythrocyte sedimentation rate (ESR), creatinine and azotemia, glycemia and complete blood count were also assessed.

#### 2.3.3. Assessment of Nutritional Status

A mini nutritional assessment (MNA) was performed for all participants. The MNA uses simple measurements and a brief questionnaire involving an anthropometric assessment (weight, height and weight loss), a general assessment (lifestyle, medication and mobility), and a dietary assessment (number of meals, food and fluid intake, self-assessment of eating autonomy and self-perception of health and nutrition). Patients ate three meals daily [28].

##### Dietary Schedule

Patients ate three meals daily, with breakfast between 07:00 and 08:00 a.m., lunch between 12:00 and 1:00 pm and dinner between 6:00 and 7:00 pm. Food intake was based on a balanced diet (with standard caloric and macro- and micronutrient content) provided by the hospital kitchen, which consisted of a repeating 4-week rotating menu, so the diet remained similar throughout the study. A trained dietitian used a calibrated dietetic spring scale to weigh all foods served and returned for three consecutive days at the beginning and end of the study. Nurses who served any food to the participants between meals recorded the amount eaten in household measurements. A computer program (DR3 v3.1.0; Sintesi Informatica, (Milano, Italy) was used to calculate the energy and the macronutrient content of the food consumed. On average, the daily nutritional intake of participants was: energy: 1622 ± 350 kcal; proteins: 59 ± 8 g; lipids: 54 ± 12 g; carbohydrates: 225 ± 4 g. 

#### 2.3.4. Assessment of Cognitive Status

The mini mental state examination (MMSE) is a well-validated and widely used assessment of global cognitive function. It takes approximately 10 to 15 minutes to administer and has a maximum score of 30 points, with lower scores representing poorer performance. The MMSE includes items assessing orientation, memory, attention, language and visual and spatial capabilities [29].

### 2.4. Randomization

Randomization was performed after the baseline assessment. Any variable that identified personal information was not included in the randomization process. Computer-generated random numbers were assigned to 159 participants who were then sorted and divided into four equal groups. The groups were randomly assigned to one of the four intervention groups: placebo (P) (n = 44), melatonin (M) (n = 42), essential amino acids (eAA) (n = 40), essential amino acids + melatonin (eAAM) (n = 33).

### 2.5. Intervention and Duration

The trial was randomized (NCT03784495), placebo-controlled and double-blind, using a parallel group design. The participants were randomly assigned to one of the following four daily treatments for 4 weeks:

(1) Placebo: (P)
(2) Melatonin (M) 1 mg/daily 30 min before going to sleep.
(3) Essential amino acids (eAA) 4 g/daily every morning during breakfast
(4) Essential amino acids (eAA) 4 g/daily every morning during breakfast + melatonin 1 mg/daily 30 min before going to sleep.

#### 2.5.1. Amino Acid Supplementation

Essential amino acids were provided for the participants of group 3 and 4 in the AAS every day with breakfast. Packets of powdered amino acid supplements (42.0% leucine, 14.0% lysine, 10.5% valine, 10.5% isoleucine, 10.5% threonine, 7.0% phenylalanine, and 5.5% other) were provided for the participants to be taken with water or milk, and they were instructed to take the 4-gram supplement once a day, every day for 4 weeks. A dietitian checked every day that every patient took the supplement and complied with the supplementation therapy.

#### 2.5.2. Melatonin Supplementation

Onemilligram of melatonin was given to the group for 4 weeks. The dose of melatonin was given 30 mins before going to sleep.

#### 2.5.3. Placebo Supplementation

The control group was given a placebo that consisted of an isocaloric amount of maltodextrin with the same flavor and appearance as the intervention product, as used in previous studies [3,30]. As occurred for the intervention, every day a dietitian checked that every patient took the supplement and complied with the supplementation therapy.

### 2.6. Physical Activity

A comprehensive physical fitness and muscle mass enhancement training program of moderate intensity was provided for all participants [31]. The exercise intervention was supervised by trained personnel and consisted of 20-min exercise sessions daily, 6 times/week for 8 weeks.

### 2.7. Sample Size

Sample size calculations used univariate one-factor repeated-measures analysis of variance (ANOVA) to examine significant differences in means at baseline and after 4 weeks. Intervention (a = 0.05, power = 0.80) with an effective size of 0.15 required a sample size of at least of 48 participants in each of the groups [32].

### 2.8. Statistical Analysis

All analyses were performed using Statistical Package for the Social Sciences, version 22.0 (SPSS Inc., Chicago, IL, USA). Descriptive statistics representing raw data for each of the three categories and the full sample were provided, including means, standard deviations and frequencies, where appropriate.

Following the verification of the normal distribution of continuous variables, the baseline data were analyzed and statistically compared between groups using one-way ANOVA. Variances were considered to be statistically significant for *p*-value < 0.05.

Data were analyzed with SPSS General Linear Modelling (GLM) repeated measures to test the hypothesized effects of the intervention over time. We conducted analyses within and between groups with a univariate ANCOVA measuring analyses of variance adjusted for covariates (gender, fat mass, body mass index and mini mental state examination) and intragroup analyses with repeated measures, using time (pre-[T1] and post-[T2] measurement) for each group.

## 3. Results

The descriptive characteristics of the study population at baseline are shown in Table 1. The sample included 159 subjects (117 women and 42 men) with a mean age over 80, and the cohort was gender unbalanced, with slightly more females. In particular, the placebo group included 31 women and 13 men, the melatonin group included 36 women and 6 men, the aminoacid group included 31 women and 9 men, and the aminoacid + melatonin group included 19 women and 14 men. On average, the patientwas at risk of malnutrition, as suggested by the mini nutritional assessment. Blood levels were within normal limits for all subjects. ANOVA revealed that, at baseline, the treatment groups were well-matched (with the exception of triglycerides) across all the biochemical parameters included in Table 1. As shown in Table 1, at baseline, the treatment groups were balanced in all body composition parameters (except for fat-free leg mass in women).

As showed by the between-group analysis in Table 2, compared with placebo (P) and melatonin (P) alone, both supplementation with eAA plus M and supplementation with eAA only increased fat-free mass (vs. P: +2190 g *p* < 0.01; vs. M: +2107 g *p* < 0.05 for EAAM and vs. P: +2618 g *p* < 0.05; vs. M: +2536 g *p* < 0.01 for eAA). M alone lowered albumin levels (vs. P: −0.39 g; *p* < 0.01; vs. eAA: −0.47 g; *p* < 0.01). In addition, the analysis showed lower gynoid fat in the eAA group vs. P (−2.23%, *p* < 0.05). The between-group analysis (groups × treatment × time) revealed a *p* < 0.05 for albumin and for total fat-free mass (*p* < 0.01).

The intra-group analysis showed a reduction in gynoid fat in M (−1.03%; *p* < 0.05) and in android fat in eAA (−2.72%; *p* < 0.01). No significant changes in inflammation markers or muscle strength were reported.

Intra-group analysis for albumin levels confirmed the results obtained by within-group analysis (M pre-post: −0.44 g; *p* < 0.001; eAAM pre-post: −0.34 g *p* < 0.05). There was also an increase in fat-free mass in eAA (+1794 g; *p* < 0.01), greater than in eAAM (+1015 g; *p* < 0.05), while for strength and inflammation by PCR no significant changes were reported, even if eAA showed a major effect on strength compared with other treatments.

## 4. Discussion

This is the first preliminary study in the literature to demonstrate that the intake of melatonin alone in the chosen clinical setting (sarcopenic elderly) can worsen protein metabolism. 

The within-group analysis demonstrated that the intake of melatonin alone significantly lowered albumin levels.

Moreover, another significant result of this study is that the presumed detrimental effect of melatonin on protein metabolism can be counteracted by supplementation with eAA.

As demonstrated by the between-group analysis, compared with the placebo and with melatonin alone, supplementation with eAA plus M increased fat-free mass both versus P: +2190 g and versus M: +2107 g. In line with numerous recent studies [3,29,30], we observed a positive trend towards increased fat-free mass in the eAA group as well. This study showed an interesting increase in fat-free mass, although the time frame of the intervention is very short compared to previous clinical trials, which lasted from 2 to 6 months [3,29,30,33,34].

It is important to emphasize that during the month of supplementation these subjects ate an adequate amount of protein (on average 60 grams per day, distributed as 20 grams at breakfast, 20 at lunch and 20 at dinner) and that they probably did not eat adequate protein before admission to hospital because all patients lived at home. In addition, during hospitalization these subjects performed moderate physical activity that they did not do at home. 

Both aerobic and resistance-type exercise training have been shown to improve the rate of decline in muscle mass and strength with age [35]. Progressive resistance training (PRT) is the most commonly used resistance therapy in older people, as demonstrated in a Cochrane review [36], and achieved substantial improvements in muscle fiber cross-sectional area (3–9%), muscle strength (100%) and physical performance [36,37].

Our results are seemingly in contrast with the current literature, which considers melatonin supplementation, in addition to its well-known beneficial effects on sleep and jet lag [38,39,40], to be a powerful anti-inflammatory agent in several human, in vivo and in vitro, experimental models. A number of different pharmacological targets of melatonin have been described, including iNOS, COX-2, cytokines and adhesion molecule production [10,11]. Interesting results were found in brain inflammation [41], in retinal inflammation [42], brain ischemia, brain trauma, spinal cord injury [43], diabetic neuropathy [44], liver ischemia, cerulein-induced pancreatitis [45], lung inflammation by pancreatic fluid [46] and after physical exercise [47,48].

However, it has been demonstrated that melatonin exerts an inhibitory [20,21] effect on insulin secretion. This direct effect of melatonin on β-cells was confirmed by the discovery of melatonin receptors on both clonal β-cells [49,50] and human islets [51]. Insulin is essential for the utilization of amino acids for anabolic purposes, even in the absence of carbohydrates, and therefore plays an important synergistic role in proteosynthesis [52]. The secretion of insulin following a protein meal promotes the uptake and utilization of amino acids for the synthesis of muscle protein, while counteracting the reverse process (proteolysis). However, aging has been repeatedly shown to be associated with the resistance of muscle tissue to the anabolic effects of insulin [24].

Therefore, if melatonin exerts an inhibitory [20,21] effect on insulin secretion, and because many studies suggest that amino acids and insulin play major roles in promoting postprandial protein anabolism [22,23], the hypothesis arises that the intake of melatonin could have a detrimental effect on protein metabolism through the inhibition of insulin secretion, particularly so in sarcopenia, which has repeatedly been shown to be associated with muscle tissue resistance to the anabolic effects of insulin [24].

We consider this a very interesting hypothesis, although the main limitation of our study is that we did not assess insulin in sarcopenic patients, even if the free fat mass analyzed in our study, with the combination of M and eAA, was decreased, compared with eAA alone.

Moreover, it is important to note that we decided to administer 1 gram of melatonin, the dosage permitted by Italian laws, which consider melatonin a supplement, and may therefore be an insufficient dosage to cause anti-inflammatory activity.

Another limitation was the short time frame of the intervention.

In addition, in this study we did not assay blood melatonin concentrations or first-morning-urine 6-sulfatoxymelatonin (aMT6s) levels at baseline or after the intervention, and we did not evaluate any antioxidant blood biomarkers nor any genetic analysis regarding the melatonin receptor 1B gene (MTNR1B).

This study observed a higher level of dropout mainly in the fourth group. This is a major weakness of this study because we cannot perform a final analysis. Regardless, this data is important in the clinical setting because it demonstrates that the combination of melatonin and aminoacids can cause possible side effects.

Finally, another limitation of our study is the use of routine parameters such as plasma albumin levels and muscle strength evaluated with handgrip, instead of using the gold standard for the evaluation of protein metabolism and muscle strength, such as labelled amino acid uptake or isokinetic muscle force. However, the reduction of circulating plasma albumin observed over time in the placebo group or in melatonin-treated patients is likely to be attributed to reduced liver synthesis and not to accelerated distribution from the intravascular space or increased degradation. The studied patients were in clinical and metabolic stability and performed physical activity according to a rehabilitation protocol. Moreover, the systemic inflammation level was marginal and was similar between all groups, with albumin reduction in the aAA group, which had a significant increase in baseline albuminemia. As for the redistribution of albumin between the various compartments of body fluids, patients had no sign of water retention (edema), being clinically and hemodynamically stable. Handgrip was chosen because of its easy use, even in elderly sarcopenic patients, and because it provides an indication of an individual’s overall strength. It also indicates nutritional status, physical function, and health status and it is predictive of mortality, hospital length of stay, and physical function [53].

Finally, a possible explanation of the decrease seen in free fat mass with the combination of M and eAA, compared with eAA, is that exogenous melatonin produces pharmacological effects on pre- and postprandial intestinal motility and then increases satiety, as already demonstrated in rats [54].

The action of melatonin corresponds to an inhibition of ISA and a reinforcement of the cyclic MMC pattern. 

One strong point is that we were able to assess the effects of melatonin supplementation separately from essential aminoacid supplementation.

## 5. Conclusions

In conclusion, this preliminary study demonstrates that the intake of melatonin alone in sarcopenic elderly patients tends to worsen protein metabolism. 

However, this supposed detrimental effect of melatonin on protein metabolism can be counteracted by supplementation with eAAs. In fact, the association of melatonin with eAAs increased fat-free mass.

Given the high number of elderly people who take melatonin for sleep/wake rhythm disorders, if these subjects are sarcopenic, we recommend caution in the intake of melatonin and advise supplementation with essential amino acids.

New randomized trials in sarcopenic elderly subjects are needed to confirm the results of our study, and also evaluate the hormonal status (insulin, GH, IGF-I, glucagon), the genetic analysis regarding the melatonin receptor 1B gene (MTNR1B) and the gold standard evaluation of protein metabolism and muscle strength, such as labelled amino acid uptake or isokinetic muscle force of these subjects. 

## Figures and Tables

**Table 1:** Characteristics of participants at baseline according to study groups.

Characteristics	Pmean ± SD	Mmean ± SD	eAAmean ± SD	eAAMmean ± SD	F	*p*-Value
General characteristics						
Gender	Women: 31 Men: 13	Women: 36 Men: 6	Women: 31 Men: 9	Women: 19 Men: 14	X2: 8.06	*p* < 0.05
Age (years)	81.86 ± 6.43	81.64 ± 7.04	80.55 ± 6.76	81.42 ± 8.02	0.28	0.84
Mini Nutritional Assessment (Score)	18.48 ± 3.45	18.31 ± 3.09	16.59 ± 3.79	17.93 ± 2.92	2.44	0.07
Mini Mental State Examination (Score)	21.43 ± 4.75	17.23 ± 3.38	21.70 ± 4.77	19.10 ± 6.48	4.92	*p* < 0.05
Weight (kg)	57.20 ±10.40	59.72 ±11.73	53.24 ±8.26	60.34 ±12.54	3.45	*p* < 0.05
Body Mass Index (kg/m^2^)	22.86 ± 0.63	24.04 ± 0.83	21.94 ± 0.72	24.29 ± 0.67	2.36	0.074
Biochemical parameters						
White Blood Cells (K/uL) (4.00–10.00)	7.01 ± 2.36	6.72 ± 2.09	7.58 ± 2.14	6.50 ± 1.98	1.73	0.16
Red Blood Cells (K/uL) (4.00–5.00)	4.18 ± 0.58	4.35 ± 0.65	4.20 ± 0.50	4.29 ± 0.42	0.84	0.47
Hemoglobine (g/dL) (12.0–16.0)	12.45 ± 1.51	12.88 ± 1.24	11.92 ± 1.73	12.79 ± 1.48	3.31	0.02
Hematrocrit (%)(36.0–46.0)	38.06 ± 4.27	39.07 ± 3.71	36.63 ± 4.38	38.59 ± 4.23	2.53	0.06
Iron (mcg/dL) (45–145)	67.55 ± 27.26	74.49 ± 29.63	60.87 ± 32.39	64.30 ± 25.75	1.60	0.19
Triglycerid (mmol/L) (<200)	124.98 ± 57.39	156.51 ± 115.23	126.08 ± 45.48	98.82 ± 28.31	4.02	*p* < 0.01
Cholesterol (mmol/L) (<200)	184.73 ± 48.74	211.27 ± 48.67	190.69 ± 52.20	186.82 ± 38.08	2.67	0.06
Albumine (gr/dL) (4.02–4.76)	3.61 ± 0.51	3.81 ± 0.40	3.53 ± 0.60	3.80 ± 0.41	3.20	0.03
Creatinine (mg/dL) (0.57–1.11)	0.87 ± 0.27	0.88 ± 0.28	0.85 ± 0.66	0.82 ± 0.29	0.20	0.90
Glycemia (mg/dL) (70–110)	100.38 ± 28.68	112.88 ± 47.33	97.58 ± 27.54	102.39 ± 29.22	1.51	0.22
Eritrocyte Sedimentation rate (mm/h) <15	49.78 ± 30.85	37.68 ± 25.51	45.94 ± 29.21	31.06 ± 24.86	3.30	*p* < 0.01
Reactive C-protein (mg/dL) (<0.50)	1.47 ± 3.18	0.48 ± 0.91	1.43 ± 2.50	0.88 ± 1.76	1.69	0.17
Homocysetine (micromol/L) (5–12)	19.13 ± 8.49	18.72 ± 7.39	17.33 ± 7.63	21.72 ± 16.37	0.82	0.49
DXA measurements						
Fat-free arm mass (grams)	4161.43 ± 133.57	3833.17 ± 178.25	3977.85 ± 153.06	4103.81 ± 142.38	0.85	0.47
Fat-free leg mass (grams)	11,827.98 ± 326.76	11,879.43 ± 436.07	11,922.22 ± 374.44	13,297.76 ± 348.31	4.07	0.008
Fat-free Mass (grams)	39,540.35 ± 813.94	37,775.79 ± 1086.21	39,074.55 ± 932.71	41,570.33 ± 867.63	2.76	0.044
Gynoid Fat mass (%)	36.45 ± 1.25	35.08 ± 1.67	33.61 ± 1.43	34.27 ± 1.33	0.86	0.46
Android Fat mass (%)	35.09 ± 1.82	36.83 ± 2.44	30.72 ± 2.09	30.98 ± 1.95	2.00	0.12
Fat Mass (grams)	18,272.27 ± 1317.45	19,517.44 ± 1758.14	14,916.64 ± 1509.68	17,418.56 ± 1404.34	1.53	0.21

**Table 2:** Effects of the interventions, within and between groups, on inflammation markers, body composition and strength.

Variable	Group	∆ Change within-Group Pre Post Intervention	*p*-Value Within-Group	∆ Change between Groups Pre Post Intervention	*p*-Value between Groups
Albumin (grams)				**M vs. P: −0.39 (−0.67; 0.10)**	**<0.01**
	P	−0.12 (−0.31; 0.07)	0.19	eAA vs. P: 0.08 (−0.22; 0.38)	0.59
	M	**−0.44 (−0.67; −0.20)**	**<0.001**	eAAM vs. P: −0.22 (−0.51; 0.06)	0.12
	eAA	0.15 (−0.16; 0.47)	0.29	**eAA vs. M: 0.47 (0.11; 0.82)**	**<0.01**
	eAAM	**−0.34 (−0.63; −0.05)**	**<0.05**	eAAM vs. M: 0.16 (−0.14; 0.47)	0.29
				eAAM vs. eAA: −0.30 (−0.65; 0.05)	0.09
Reactive C−protein (mg/dL)				M vs. P: 0.94 (−0.83; 2.71)	0.29
	P	−0.20 (−0.98; 0.58)	0.6	eAA vs. P: 0.72 (−1.19; 2.64)	0.46
	M	0.80 (0.88; 2.49)	0.33	eAAM vs. P: 0.75 (−1.12; 2.63)	0.43
	eAA	0.63 (−0.47; 1.72)	0.22	eAA vs. M: −0.22 (−2.29; 1.86)	0.84
	eAAM	0.65 (−0.83; 2.12)	0.35	eAAM vs. M: 0.16 (−0.14; 0.47)	0.84
				eAAM vs. eAA: −0.30 (−0.65; 0.05)	0.98
Fat−free arm mass (grams)				M vs. P: 333.83 (−113.85; 781.52)	0.14
	P	0.34 (−0.46; 1.15)	0.38	eAA vs. P: 397.80 (−73.99; 869.60)	0.1
	M	−0.67 (−1.47; 0.24)	0.09	eAAM vs. P: 424.26 (−41.08; 889.59)	0.07
	eAA	0.45 (−0.18; 1.08)	0.12	eAA vs. M: 63.97 (−445.36; 573.31)	0.8
	eAAM	0.29 (−0.15; 0.72)	0.17	eAAM vs. M: 90.42 (−388.49; 569.33)	0.71
				eAAM vs. eAA: 26.45 (−495.81; 548.71)	0.92
Fat−free leg mass (grams)				M vs. P: −230.59 (−972.52; 511.33)	0.54
	P	0.14 (−0.35; 0.64)	0.55	eAA vs. P: 393.99 (−373.81; 11171.79)	0.31
	M	−0.06 (−0.56; 0.44)	0.81	eAAM vs. P: 194.67 (−578.33; 967.67)	0.62
	eAA	**0.35 (0.03; 0.67)**	**<0.05**	eAA vs. M: 629.58 (−200.42; 1459.59)	0.14
	eAAM	0.04 (−0.99; 1.06)	0.94	eAAM vs. M: 425.26 (−353.71; 1201.23)	0.28
				eAAM vs. eAA: −204.32 (−1056.06; 647.42)	0.64
Fat−free Mass (grams)	P	−233.73 (−1081.62; 614.16)	0.58	M vs. P: 82.16 (−1461.35; 1625.68)	0.92
		807.72 (−210.07; 1825.52)	0.11	**eAA vs. P: 2618.49 (978.23; 4258.74)**	**<0.05**
	M	**1794.17 (783.29; 2805.05)**	**<0.01**	**eAAM vs. P:2190.01 (555.16; 3824.87)**	**<0.01**
		**1015.45 (139.65; 1891.25)**	**<0.05**	**eAA vs. M: 2536.33 (766.13; 4306.53)**	**<0.01**
	eAA			**eAAM vs. M: 2107.85 (436.45;** 3779.25)	**<0.05**
				eAAM vs. eAA: −428.48 (−2275.11; 1418.16)	0.65
	eAAM				
Gynoid Fat mass (%)				M vs. P: −1.60 (−3.78; 0.59)	0.15
	P	0.01 (−1.87; 1.89)	0.99	**eAA vs. P: −2.23 (−4.49; 0.04)**	**<0.05**
	M	**−1.03 (−2.05; −0.01)**	**<0.05**	eAAM vs. P: −0.76 (−3.03; 1.51)	0.51
	eAA	−1.54 (−3.51; 0.42)	0.11	eAA vs. M: −0.63 (−3.08; 1.82)	0.61
	eAAM	−0.33 (−1.85; 1.20)	0.66	eAAM vs. M: 0.84 (−1.53; 3.20)	0.48
				eAAM vs. eAA: 1.47 (−1.09; 4.02)	0.26
Android Fat mass (%)				M vs. P: 0.53 (−2.20; 3.27)	0.7
	P	−1.29 (−3.84; 1.25)	0.3	eAA vs. P: −1.51 (−4.39; 1.36)	0.3
	M	−1.07 (−2.60; 0.46)	0.16	eAAM vs. P: 0.42 (−2.48; 3.33)	0.77
	eAA	**−2.72 (−4.77; −0.68)**	**<0.01**	eAA vs. M: −2.04 (−5.16; 1.08)	0.2
	eAAM	−0.04 (−2.23; 2.15)	0.97	eAAM vs. M: −0.11 (−3.07; 2.85)	0.94
				eAAM vs. eAA: 1.93 (−1.33; 5.19)	0.24
Handgrip Strength (Kg)				M vs. P: −0.37 (−2.52; 1.77)	0.73
	P	−0.11 (−1.40; 1.40)	1	eAA vs. P: 0.98 (−1.37; 3.33)	0.41
	M	0.09 (−1.77; 1.95)	0.92	eAAM vs. P: 0.04 (−2.07; 2.15)	0.97
	eAA	0.15 (−1.36; 1.66)	0.82	eAA vs. M: 1.36 (−0.97; 3.68)	0.25
	eAAM	−0.06 (−1.54; 1.43)	0.94	eAAM vs. M: 0.42 (−1.61; 2.44)	0.68
				eAAM vs. eAA: −0.94 (−3.27; 1.39)	0.42

in **bold** *p* value <0.05 and <0.01.
